# Emotional Appetite Questionnaire: psychometric properties in Brazilian adult samples before and after the COVID-19 pandemic onset

**DOI:** 10.7717/peerj.14597

**Published:** 2023-01-26

**Authors:** Bianca G. Martins, Nadine V. Vanini, Lucas A. Campos, Juliana ADB Campos

**Affiliations:** 1 Department of Biological Sciences, School of Pharmaceutical Sciences, Universidade Estadual Paulista, Araraquara, São Paulo, Brazil; 2 Faculty of Medicine and Health Technology, University of Tampere, Tampere, Finland; 3 Tampere University Hospital, Tampere, Finland; 4 Dental Sciences Graduate Program, School of Dentistry, São Paulo State University (UNESP), Araraquara, São Paulo, Brazil

**Keywords:** Appetite, Psychometrics, Eating behavior, Emotions, Food, Appetite control

## Abstract

**Background:**

Appetite represents a desire of a person to eat specific food in order to reach satisfaction and pleasure states. This desire may be associated with the experience of negative or positive emotions (emotional appetite). Emotional appetite can influence eating behavior, and its investigation is relevant to avoid possible damage to health resulting from a disordered eating.

**Objectives:**

To adapt the Emotional Appetite Questionnaire (EMAQ) to the Portuguese language; to assess the validity and reliability of the data; and to assess emotional appetite in three samples of adults collected before and after the outbreak of the COVID-19 pandemic.

**Methods:**

This is a cross-sectional study with non-probabilistic convenience sampling. The Portuguese version of the EMAQ was presented after translation, back-translation, and content analysis. Two studies were conducted, the first before and the second after the pandemic onset. Three samples were formed (2019: Sample 1 (age = 19.7 ± 1.5 years) *n* = 323; 2020: Sample 2 (age = 21.3 ± 1.8 years) *n* = 1,011; and Sample 3 (age = 28.9 ± 3.1 years) *n* = 909). An exploratory strategy with parallel analysis was performed. The analyses were conducted in FACTOR and R (lavaan and semTools packages) software. After determining the best-fit model for the data, emotional appetite was examined considering decrease, non-alteration, and increase in appetite in the face of positive and negative emotions/situations. The profile of emotional appetite was determined using a circumplex model.

**Results:**

The two-factor model described by the valence of emotions/situations fitted the samples (Comparative Fit Index_minimum-maximum_ = 0.95–0.98; Tucker-Lewis Index = 0.94–0.98; Root Mean Square Error of Approximation = 0.03–0.08; α_ord_ = 0.78–0.88). Increases in appetite were more frequent for positive emotions/situations (52.0–57.5%), and both decreases (35.4–44.5%) and increases (50.0–56.2%) in appetite were observed for negative emotions/situations. Emotions with negative valence and activation were more relevant to appetite reduction, while a significant increase in appetite was observed with anxiety (negative valence and positive activation).

**Conclusion:**

Different emotions and situations may influence appetite in people, and such an investigation may be useful in preparing eating protocols.

## Introduction

Appetite is the desire of a person to eat a certain food in order to reach a state of satisfaction (Alvarenga & Koritar, 2019). This desire may lead to the consumption of certain food groups (Alvarenga, 2015), and the choice may be associated with the experience of a negative or positive emotions (emotional appetite) (Evers et al., 2013; Lindeman & Stark, 2001; Macht, 2008). The effects of negative emotions for eating behavior have been studied extensively over time (Alvarenga & Koritar, 2019; Canetti, Bachar & Berry, 2002; Lindeman & Stark, 2001; Macht, 2008; Martins et al., 2020a; Van Strien et al., 2013), whereas positive emotions have only recently come into focus (Evers et al., 2013; Macht, 1999, 2008; Van Strien et al., 2013). Some authors suggest that the valence of emotions is an important aspect in the assessment of emotional eating, as individuals may behave and express their appetite differently in the face of different emotions (Macht, 1999, 2008; Nolan, Halperin & Geliebter, 2010; Van Strien et al., 2013). Geliebter & Aversa (2003) add that examining emotional appetite as a result of routine situations is also relevant because these situations may trigger emotions that could also change eating behavior (emotional eating) (Alvarenga, 2015; Canetti, Bachar & Berry, 2002; Karlsson et al., 2000). Emotional appetite then plays an important role not only by altering habitual food intake, but also by representing a way of dealing with one’s emotions (Canetti, Bachar & Berry, 2002).

Psychometric instruments such as the Dutch Eating Behavior Questionnaire (DEBQ) (Van Strien et al., 1986), the Three-Factor Eating Questionnaire (TFEQ-18) (Karlsson et al., 2000) and the Emotional Eating Scale (EES) (Arnow, Kenardy & Agras, 1995) can be used to assess appetite and emotional eating behavior. However, these scales only consider the influence of negative emotional aspects on eating and focus on the eating behavior itself. Given the emphasis on negative emotions and the need to include the evaluation of routine situations related to the eating process, Geliebter & Aversa (2003) suggested using the Emotional Appetite Questionnaire (EMAQ) to examine emotional appetite and determine the role of negative and positive emotions and situations in changing individual eating behavior.

Although the EMAQ is an interesting instrument, it has been little used in the literature (Bourdier et al., 2017; Geliebter & Aversa, 2003; Nolan, Halperin & Geliebter, 2010; Sabry et al., 2020) compared to other instruments such as TFEQ-18 (Brytek-Matera, Rogoza & Czepczor-Bernat, 2017; Karlsson et al., 2000; Lauzon et al., 2004; Martins et al., 2020b; Medeiros et al., 2017; Stunkard & Messick, 1985) and DEBQ (Arhire et al., 2021; Doostfatemeh et al., 2022; Małachowska, Jeżewska-Zychowicz & Gębski, 2021; Van Strien et al., 1986). One plausible reason for this is the complexity of assessing emotions, especially when negative and positive aspects are examined simultaneously (Russell, 1980). In the context of emotions, the study of valence has not been comprehensive enough to account for the nuances of the subject. For example, emotion can also have a potential contribution to behavior by predisposing an action (activation of emotion) (Crispim et al., 2017; Russell, 1980). Moreover, both appetite and eating behaviors are multidimensional concepts with individual, social, and environmental influences (Alvarenga, 2015), making their assessment complex. The investigation of possible determinants of eating behavior has gained importance in the literature (Barnhart, Braden & Price, 2021; Mason, Barrington-Trimis & Leventhal, 2021), especially with regard to individual components. The reason is that inadequate consumption of food in terms of quantity and quality can be detrimental to health (Alvarenga, 2015; Alvarenga & Koritar, 2019). In this sense, understanding the aspects that precede and influence food consumption becomes relevant. This can be important for health professionals to effectively assess and address one’s food needs. Thus, examining the role of emotion in an individual’s desire to consume certain foods is a task that is as challenging as it is important.

The EMAQ is available in the original English version (Geliebter & Aversa, 2003; Nolan, Halperin & Geliebter, 2010) and in versions adapted to the French (Bourdier et al., 2017) and Brazilian (Sabry et al., 2020) contexts. Sabry et al. (2020) validated the Portuguese version of the EMAQ in a Brazilian sample in 2020. However, this version presents some inconsistencies when compared to the original version (Geliebter & Aversa, 2003; Nolan, Halperin & Geliebter, 2010). These inconsistencies are mainly found in the translation of some items such as “Bored”, “Angry”, “Frustrated” and “After a heated argument”. The content of these items may be interpreted slightly differently than expected in the Brazilian and other Lusophone populations. Thereby, we included the cultural adaptation of the instrument for Portuguese as an objective of this study. This new version will ensure, after the psychometric properties have been verified, that the EMAQ is measuring adequately and in a standardized way the emotional appetite as originally proposed (Geliebter & Aversa, 2003).

The study of emotional appetite proposed by Geliebter & Aversa (2003) offers two alternatives for analyzing information. The first considers the effects of emotions and situations and their valences separately (four-factor model: negative emotions, positive emotions, negative situations, and positive situations). The second emphasizes only the valence of the event (two-factor model: emotions and situations: negative *vs*. positive). These models have been tested in traditional analytic approaches, mainly using exploratory factor analysis, to demonstrate the validity of the internal structure of the EMAQ for different samples (Bourdier et al., 2017; Sabry et al., 2020). As a result of these studies, the two-factor model has been widely accepted. Bourdier et al. (2017) applied the EMAQ to French university students and found that the data showed an acceptable fit to the two-factor model (RMSEA = 0.08; SRMR = 0.07; CFI = 0.83), although some items had low factor loadings (scared = 0.37; in love = 0.34). Sabry et al. (2020) examined the internal structure of the EMAQ in Brazilian women using the public health system and also found retention of two factors. In addition, the data obtained with the EMAQ was reliable (Bourdier et al., 2017; Sabry et al., 2020). Bourdier et al. (2017) found that the positive and negative factors of the EMAQ showed Cronbach’s α = 0.75 and 0.85 respectively. Sabry et al. (2020) observed that the same factors showed α = 0.79 and 0.87, respectively. Both studies found that individuals reduced their food consumption in the face of negative emotions/situations.

Furthermore, the inclusion of theoretical models of emotions evaluation could enrich the interpretation of the data obtained with the EMAQ. It will also allow a more comprehensive discussion and understanding of emotional appetite. Crispim et al. (2017) relied on Russell’s circumplex model (Russell, 1980) to better understand people’s emotional experiences. This model assesses simultaneously the valence (negative x positive) and activation (calm x aroused) of emotions in a bipolar (each dimension has two extremes) and orthogonal (independence between dimensions) proposal (Crispim et al., 2017; Russell, 1980). This model may be of interest for the analysis of emotional appetite, as it allows an interpretation that goes beyond the traditional binomial approach of negative and positive emotions.

The literature discusses the susceptibility of certain groups, such as young adults, to inappropriate eating behaviors (Bourdier et al., 2017; Martins et al., 2020a, 2020b; Nelson et al., 2008). Nelson et al. (2008) mention that this age group is in a phase of life where new responsibilities are assumed and control is taken over daily routines, including meal selection and preparation. In addition, the new experiences of autonomy and freedom may involve emotional demands that are often difficult to process because of the possible immaturity of the cognitive repertoire and mental abilities related to emotions that are prevalent in young people (Nelson et al., 2008; O’Brien, 2015). In this context, individuals may attempt to alter their emotional appetite by seeking relief or satisfaction in the face of negative/positive emotions/situations or feelings of elation and social integration (Martins et al., 2020a), which may lead to changes in habitual eating behavior. These changes in eating behaviors may jeopardize the health of these people if they become entrenched over time.

In addition to the greater vulnerability of young people, context may also influence the propensity for emotional appetite and eating. Currently, COVID-19 may be considered a stressor (Campos et al., 2021; Mason, Barrington-Trimis & Leventhal, 2021), both because of the disease itself and because of the disruption of personal routines and recommended social isolation to prevent the spread of the virus. In this context of uncertainty, individuals may change their food consumption because of the tensions they experience (Mason, Barrington-Trimis & Leventhal, 2021). In Mason, Barrington-Trimis & Leventhal (2021) study of young adults, 31.0% of participants reported changing their diet during the pandemic. Poelman et al. (2021) found changes in appetite in Dutch adults, and during the lockdown, the prevalence of reduction (8.2%) and increase (8.9%) in food consumption was similar. In addition, these authors (Poelman et al., 2021) found that younger adults were more likely to report both an increase (odds ratio (OR) = 1.61; 95% confidence interval 95% CI [1.01–2.57]) and a decrease (OR = 1.02; 95% CI [1.01–1.04]) in food consumption compared with older adults. In light of the above, it is concluded that monitoring emotional eating in these two conditions (age group and context) may be important to help health professionals and researchers develop interventions that support the adoption of healthy eating and emotion regulation habits. It also highlights the need for further investigations of emotional appetite in the post-pandemic context, to track any changes that may contribute to the adoption of unhealthy eating habits.

Given all the above, we conducted this study to answer two questions: (1) Does the EMAQ present acceptable psychometric indicators when applied to samples of Brazilian adults of different age groups before and after the beginning of the COVID-19 pandemic? (2) Does emotional appetite change when facing different emotions/situations for these different samples? Thus, the aims of the present study were to (i) transculturally adapt the Emotional Appetite Questionnaire (EMAQ) for the Portuguese language, (ii) assess the validity and reliability of the data collected with the EMAQ, and (iii) evaluate emotional appetite in three samples of Brazilian adults collected before and after the pandemic outbreak.

## Materials and Methods

### Instrument

The EMAQ was originally developed in English by Geliebter & Aversa (2003) to assess emotional appetite. The instrument contains 22 items, 14 of which relate to emotions (nine negative and five positive emotions) and eight relate to everyday situations (five negative and three positive situations). The EMAQ items ask whether individuals reduce, maintain, or increase their food intake in the face of emotions and/or situations. The response scale ranges from 1 to 9, with scores from 1 to 4 representing a decrease in appetite, five representing the neutral point (no change), and scores from 6 to 9 representing an increase in appetite. EMAQ is an instrument of public use.

As mentioned earlier, emotional appetite in the original proposal of the EMAQ can be assessed using two models: 1. Four-factor model (negative emotions, positive emotions, negative situations, and positive situations); 2. Two-factor model (negative *vs*. positive emotions and situations) (Geliebter & Aversa, 2003). It should be noted, however, that these propositions were conducted on a sample of the North American population categorized as underweight, normal weight, and overweight, with a mean age ranging from 28.9 to 33.5 years.

The evaluation of the validity and reliability of the data collected with the EMAQ in our study was performed considering the recommendations of the Standards for Educational and Psychological Testing (American Educational Research Association (AERA), American Psychological Association (APA), National Council on Measurement in Education (NCME), 2014). Thus, content validity and internal and external validity were estimated in this work.

### Elaboration of the EMAQ’s Portuguese version and evidence of validity based on test content

The EMAQ was translated into Portuguese following the orthographic agreement between Portuguese-speaking countries aiming to obtain a version that is suitable for different Lusophone countries and contexts. The translation into Portuguese was done by three independent, bilingual translators (two Portuguese and one Brazilian). These three versions were compared with each other and with the English version by the first (BGM) and last (JADBC) authors of this study to obtain a consensual Portuguese version. Subsequently, a back-translation was performed by two translators whose native language was English. The back translators were not informed that this was a back translation process. Subsequently, a team of six experts in eating behavior and psychology assessed the linguistic, cultural, idiomatic, and conceptual equivalence of the final version instrument (Content Validity Index–CVI) and we expected 100% equivalence, which was confirmed. All analyses were performed independently by the specialists as recommended by Beaton et al. (2007). The instrument (Table 1) was pretested in a group of 25 young adults (mean age: 20.4 years [standard deviation—SD = 2.8]) with similar characteristics (as weight status) to those expected for the study sample. This pre-test was to verify the Misunderstanding Index (MI) of the items, and no item had an MI > 20% (MI_minimum-maximum_ = 0.0–1.7%), which is considered adequate.

**Table 1:** Original and Portuguese versions of the Emotional Appetite Questionnaire (EMAQ).

Original version*		Portuguese version	
**The following refers to EMOTIONS.** As compared to usual, do you eat:		**As afirmações seguintes descrevem EMOÇÕES**. Comparativamente ao usual, você come:	
When you are	1. Sad	Quando você está	1. Triste
	2. Bored		2. Aborrecido^#^
	3. Confident		3. Confiante
	4. Angry		4. Zangado^#^
	5. Anxious		5. Ansioso
	6. Happy		6. Feliz
	7. Frustrated		7. Frustrado^#^
	8. Tired		8. Cansado
	9. Depressed		9. Deprimido
	10. Frightened		10. Assustado
	11. Relaxed		11. Relaxado
	12. Playful		12. Brincalhão
	13. Lonely		13. Solitário
	14. Enthusiastic		14. Entusiasmado^#^
**The following refer to SITUATIONS** As compared to usual, do you eat:		**As afirmações seguintes referem-se às SITUAÇÕES**. Comparativamente ao usual, você come:	
15. When under pressure		15. Quando está sob pressão	
16. After a heated argument		16. Depois de uma discussão forte^#^	
17. After a tragedy of someone close to you		17. Depois de uma tragédia que aconteceu a alguém próximo	
18. When falling in love		18. Quando se apaixona	
19. After ending a relationship		19. Quando termina uma relação^#^	
20. When engaged in an enjoyable hobby		20. Quando se envolve com um passatempo agradável	
21. After losing money or property		21. Depois de perder bens ou dinheiro	
22. After receiving good news		22. Depois de receber boas notícias	

**Notes:**

* Geliebter A, Aversa A. Emotional eating in overweight, normal weight, and underweight individuals. Eating Behaviors. 2003; 3(4): 341-7.

# These items are slightly different from those presented in recent Portuguese version of EMAQ published in Brazilian context: 2: sem nada para fazer (nothing to do); 4: Com raiva (Angry); 7: Decepcionado (disappointed); 14: Animado (lively); 16: Depois de uma grande discussão (after a big discussion) 19: Após o fim de um relacionamento (After the ending of a relationship) (Source: Sabry et al. (2020)).

After developing the Portuguese version, we investigated the internal and external validity of the EMAQ. To this end, two studies were conducted, one before the pandemic (Study 1) and one after the start of the pandemic (Study 2), which are described below.

### Study design, participants and recruitment

This was a cross-sectional study with a convenience (non-probability) sample. The minimum sample size was calculated from Westland’s (2010) proposal. This recommendation was based on the expected effect size (EEsize), number of latent (L) and manifest (M) variables, significance level adopted (α), and analytical power (β). Thus, for the 4-factor EMAQ model, we considered EEsize = 0.30, L = 26 (factors + item errors), M = 22, α = 0.05 and β = 0.80, which resulted in the need for 241 participants. For the 2-factor EMAQ model, we considered EEsize = 0.30, L = 24, M = 22, α = 0.05 and β = 0.80, which resulted in the need for 237 participants. Considering that 241 respondents would be sufficient to achieve an adequate power of analysis for both models, and a loss rate of 15% (*e.g*., individuals who could not respond to all EMAQ items), the minimum sample size was 284 respondents.

The first data collection (Study 1) was conducted before the pandemic, between March and July 2019. Students enrolled in a public university were asked to complete the EMAQ (Sample 1) and a sample characterization instrument. The instruments were paper-based and self-completed in dedicated rooms. Researchers informed that participation was voluntary and anonymous. The inclusion criterion was age between 18 and 35 years. Individuals with diabetes, dietary restrictions, and pregnant women were not included because they might have certain characteristics that could alter their emotional appetite. All participants signed the informed consent form presented after the researcher explained the research objectives and clarified that participation was voluntary and anonymous. The total number of participants in study one was 391 adults, however only those who completed all items of the EMAQ were considered eligible, resulting in 323 participants (response rate = 82.6%). Study 1 was approved by the Ethics Committee for Research on Human Beings of the School of Pharmaceutical Sciences (FCF) (CAAE: 67409517.0.0000.5426).

The second data collection was conducted after the onset of the pandemic in Brazil (Study 2), from November 2020 to March 2021. In this scenario, data collection had to be adapted to the remote context and was conducted online using Google Forms. The consent form was presented to the participants before starting the research, so that consent was obtained by selecting an option whose content referred to the agreement to participate in the study. The EMAQ and sample characterization instrument were completed by the participants themselves. A non-probability sampling design was chosen, and the research was disseminated by the snowball technique (each participant indicated new participants). Recruitment began among undergraduate students and each participant was asked to share the link with their friends, and social network contacts (Parker, Scott & Geddes, 2019). Only individuals between the ages of 18 and 35 (young adults) were eligible to participate in the study (inclusion criterion). Individuals with diabetes, dietary restrictions, and pregnant women were not included because they might have certain characteristics that could alter their emotional appetite. After completing data collection and before performing statistical analyses, the sample was divided into two age groups: 18 to 24 years (Sample 2) and ≥25 years (Sample 3). The division by age group was based on the literature (Nelson et al., 2008) and on the characteristics of the participants in Study 1 to allow comparison of the results between the samples and to exclude the variable age as a possible confounding factor. The total number of participants in Study 2 was 2,166 adults, however only those who completed all items of the EMAQ were considered eligible, resulting in 1,920 participants (response rate = 88.6%). Study 2 was approved by the Ethics Committee for Research on Human Beings of the School of Pharmaceutical Sciences (FCF) (CAAE: 38041520.1.0000.5426).

Both studies collected information on sex, age (in years), weight (kg), height (m), and economic level. Self-reported values for weight (kg) and height (m) were used to calculate body mass index (BMI) and classify anthropometric weight status based on the reference values established by the WHO (2000). Economic level was assessed by participant-reported monthly family income ($0 to $389.41; $389.42 to $1,678.91; and ≥$1,678.92) and classified as low, medium, and high.

Participants of Study 2 (during the pandemic) were asked whether they perceived changes in their own diet after the onset of the pandemic (no, yes), whether there was a change in the amount of food consumed during this period (decreased, not decreased nor increased, increased), and whether there was an influence of emotions on their eating (no, yes).

After applying the EMAQ to each sample, the validity (based on the internal structure and compared to the external measure) of the data obtained was examined.

### Evidence of validity based on the internal structure

First, the psychometric sensitivity of the EMAQ items was tested in the samples. To do this, measures of summary and the shape of the distribution of responses to the items were used. Absolute skewness and kurtosis values of less than three and seven, respectively, indicated that there was no serious violation of the normality assumption (Kline, 2016).

In the absence of conclusive evidence to operationalize the EMAQ in a sample of young Brazilian adults, it was decided to perform an exploratory factor analysis to extract four and two factors, respectively, as recommended by Geliebter & Aversa (2003) and Bourdier et al. (2017). The Diagonal Weighted Least Square (DWLS) estimator was used to extract the factors, considering the number of points on the response scale (nine points—polychoric matrix). The adequacy of the data for factor analysis was checked with the Kaiser-Meyer-Olkin index (KMO) and was considered adequate if it was ≥0.80 (Marôco, 2021).

After checking the assumptions, parallel analysis with optimized procedure and Direct Oblimin rotation, which uses bootstrap procedures and comparison of results with real data with Monte Carlo simulations, were used to recommend the number of factors to be retained (Jennrich & Sampson, 1966; Timmerman & Lorenzo-Seva, 2011). Diagonal Weighted Least Square (DWLS) was used as the estimation method, and the fit of the models to the data was assessed using the Comparative Fit Index (CFI), Tucker-Lewis Index (TLI), and Root Mean Square Error of Approximation (RMSEA), with CFI and TLI ≥0.90 and RMSEA ≤0.10 considered reasonable (Kline, 2016). To examine whether the matrix underlying the EMAQ data showed signs of unidimensionality, the indices Unidimensional Congruence (UniCo), the Explained Common Variance (ECV), and the Mean of Item Residual Absolute Loadings (MiReal) were used. Values of UniCo ≥0.95, ECV ≥0.85, and MiReal ≤0.30 were considered to indicate unidimensionality (Ferrando & Lorenzo-Seva, 2018). The stability of the factors extracted in the factor analysis was assessed using the H index, which is considered appropriate when H >0.80 (Ferrando & Lorenzo-Seva, 2018). The factor loadings of the items were analyzed in the rotated matrix, and items with loadings ≤0.40 were excluded from the model (refinement process). These analyses were performed using the program FACTOR 11.04.02 (Lorenzo-Seva & Ferrando, Tarragona, Spain) (Ferrando & Lorenzo-Seva, 2017; Lorenzo-Seva & Ferrando, 2006).

Convergent construct validity of the EMAQ factors was examined using the average variance extracted (AVE) and was considered adequate when AVE ≥0.50 (Fornell & Larcker, 1981). Because the EMAQ model has more than one factor, discriminant validity was also assessed when AVE_F1_ and AVE_F2_ ≥ r²_(F1×F2)_ (Fornell & Larcker, 1981).

The reliability (internal consistency) of each factor was estimated using composite reliability (CR) and ordinal coefficient alpha (α_ord_), with values of CR and α_ord_ ≥0.70 considered appropriate (Kline, 2016).

After determining the factorial solution of the EMAQ that best fitted the data, the invariance between samples was estimated (sample 1 *vs*. 2; 2 *vs*. 3; 1 *vs*. 3). Multigroup analysis was performed using the CFI difference method (ΔCFI) (Cheung & Rensvold, 2002). In this context, a series of nested models were evaluated, namely the configural (M0), metric (M1), scalar (M2), and strict (M3) models. For confirmation of metric invariance, ΔCFI_M1–M0_ should be < |0.01|. Scalar and strict invariance were attested when ΔCFI_M2–M1_ < |0.01| and ΔCFI_M3–M2_ < |0.01|, respectively (Cheung & Rensvold, 2002). The assessment of invariance is useful to determine whether the model performs in the same way in the different samples. If invariance is confirmed, inferences and direct comparisons of the results obtained are possible (Kline, 2016). The investigation of convergent and discriminant construct validity, reliability and factor invariance were performed using the R program v. 4.2.1 (R Core Team, 2022) with the lavaan v. 0.6-12 (Rosseel, 2012) and semTools v. 0.5-6 (Jorgensen et al., 2018) packages.

### Evidence of validity based on relations to other variables

The validity of the EMAQ factors was also investigated in relation to the Emotional Eating factor of the Three-Factor Eating Questionnaire (TFEQ-18) (Karlsson et al., 2000), using the polychoric correlation coefficient. A Portuguese version of the TFEQ-18, developed by Martins et al. (2020b), which had a good fit to the data, was used (confirmatory factor analysis: Sample 1: CFI = 0.961; TLI = 0.955; RMSEA = 0.057; Sample 2: CFI = 0.928; TLI = 0.917; RMSEA = 0.079; Sample 3: CFI = 0.943; TLI = 0.934; RMSEA = 0.074). A strong and significant correlation (convergent positive validity) of the factor related to negative emotions/situations of EMAQ with emotional eating is expected, and a weak and negative correlation (convergent negative validity) with the factor related to positive emotions/situations of EMAQ due to the proposal construct theory. These procedures were performed using the R program v. 4.2.1 (R Core Team, 2022) with the lavaan v. 0.6-12 (Rosseel, 2012) and semTools v. 0.5-6 (Jorgensen et al., 2018) packages.

After confirming the validity and reliability of the data obtained with the EMAQ, both in terms of internal structure and external measure, the emotional appetite of the samples was assessed.

### Investigation of emotional appetite

First, participants were categorized as decreasing (mean score < 5.00), unchanged (score = 5.00), and increasing (score > 5.00) appetite in the face of positive and negative emotions/situations. The prevalence of these categories was calculated with a 95% confidence interval (95% CI). The prevalence of appetite changes was also examined, with each EMAQ item considered separately. Comparison of prevalence between samples was performed with the z test (α = 5%).

The profile of emotional appetite in the three samples was also assessed using the circumplex model, in which only the EMAQ items related to emotions were considered. In constructing this model, two groups were considered: those who reported decreased appetite and those who reported increased appetite in the face of negative and positive emotions (Geliebter & Aversa, 2003).

## Results

Samples 1, 2, and 3 consisted of 323, 1,011, and 909 participants, respectively. The mean age of participants in Sample 1 was 19.7 years (SD = 1.5), in Sample 2 was 21.3 years (SD = 1.8), and in Sample 3 was 28.9 years (SD = 3.1). Information about the samples is shown in Table 2.

**Table 2:** Characterization of the three study samples.

	^‡^Sample 1	Sample 2	Sample 3
**Characteristic** ^§^	***n* (%)**	***n* (%)**	***n* (%)**
**Sex**			
Male	80 (24.8)	238 (23.7)	252 (27.8)
Female	243 (75.2)	763 (76.0)	654 (72.2)
Non-binary	–	3 (0.3)	–
**Anthropometric weight status**			
Low weight (<18.5 kg/m²)	23 (7.3)	86 (8.7)	20 (2.2)
Normal range ([18.5–25.0] kg/m²)	213 (67.1)	648 (65.1)	498 (55.8)
Overweight ([25.0–30.0] kg/m²)	70 (22.1)	168 (16.9)	235 (26.3)
Obesity (≥30.0 kg/m²)	11 (3.5)	92 (9.3)	140 (15.7)
**Economic level (monthly average income)** **^#^**			
Low income ($0 to $389.41)	7 (2.2)	259 (25.9)	145 (16.1)
Medium income ($389.42 to $1,678.91)	123 (38.1)	492 (49.1)	525 (58.1)
High income (above $1,678.92)	193 (59.7)	251 (25.0)	234 (25.8)
**In the face of the pandemic, do you feel that there has been a change in your diet?**			
No	–	135 (13.4)	181 (19.9)
Yes	–	875 (86.6)	727 (80.1)
**In the context of the pandemic, do you feel that the amount of food ingested:**			
Decreased	–	216 (21.4)	139 (15.1)
Neither decreased nor increased	–	290 (28.7)	337 (37.1)
Increased	–	505 (49.9)	433 (47.8)
**Do you believe that your emotions influenced your eating habits in the context of the COVID-19 pandemic?**			
No	–	63 (6.2)	82 (9.0)
Yes	–	948 (93.8)	827 (91.0)

**Notes:**

‡ Sample 1: *n* = 323, 18–24 years old, before the pandemic; Sample 2: *n* = 1,011, 18–24 years old, after the start of the pandemic; Sample 3: *n* = 909, 25 years or older, after the start of the pandemic.

§ Not all participants answered the sample characterization questions.

# Brazilian Reals (BRL) were converted into American dollars (exchange rate in June 2022–1 dollar ($) = 5.15 BRL–available in https://www.bcb.gov.br).

Regarding the psychometric sensitivity of the EMAQ items based on the minimum and maximum, it was found that for items 10 (Frightened) and 22 (After receiving good news), not all possible response categories were represented and there was a strong bias for responses with lower scale values, which affected the normality of the data. After removing these items, the model was labeled “initial”, and the psychometric sensitivity of the other EMAQ items (samples 1, 2, and 3) was adequate, *i.e*., there was no serious violation of the normality assumption. More information about the descriptive statistics of the answers given to the EMAQ items can be found in Table 3.

**Table 3:** Descriptive statistics on the answers given to the items of the Emotional Appetite Questionnaire instrument by the participants.

^#^Sample 1/Sample 2/Sample 3							
**Item**	**Mean**	**Median**	**Standard deviation**	**Skewness**	**Kurtosis**	**Minimum**	**Maximum**
EMAQ1	5.22/4.86/5.10	5.00/5.00/5.00	2.45/2.46/2.43	−0.14/−0.02/−0.08	−1.11/−1.10/−0.98	1/1/1	9/9/9
EMAQ2	5.03/4.95/5.21	5.00/5.00/5.00	2.00/2.11/2.11	0.15/−0.01/−0.22	−0.56/−0.52/−0.43	1/1/1	9/9/9
EMAQ3	5.03/5.00/5.03	5.00/5.00/5.00	1.16/1.22/1.31	−0.27/−0.40/−0.43	3.70/3.28/3.08	1/1/1	9/9/9
EMAQ4	4.76/4.79/4.92	5.00/5.00/5.00	1.88/1.95/1.88	−0.06/−0.03/−0.09	0.03/−0.10/0.25	1/1/1	9/9/9
EMAQ5	6.47/6.21/6.53	7.00/7.00/7.00	2.57/2.67/2.45	−0.89/−0.73/−0.92	−0.36/−0.72/−0.14	1/1/1	9/9/9
EMAQ6	5.69/5.62/5.58	5.00/5.00/5.00	1.44/1.40/1.44	0.14/−0.13/−0.10	1.44/1.79/1.78	1/1/1	9/9/9
EMAQ7	5.27/5.41/5.59	5.00/5.00/5.00	2.00/2.18/2.11	−0.12/−0.05/−0.21	−0.43/−0.70/−0.43	1/1/1	9/9/9
EMAQ8	4.23/4.62/4.85	4.00/5.00/5.00	1.79/2.05/2.03	0.30/0.28/0.09	0.03/−0.16/−0.21	1/1/1	9/9/9
EMAQ9	4.75/4.42/4.75	5.00/4.00/5.00	2.51/2.58/2.48	0.10/0.29/0.10	−1.08/−1.07/−0.98	1/1/1	9/9/9
EMAQ10	3.84/3.99/4.15	5.00/5.00/5.00	1.64/1.78/1.83	−0.44/−0.05/0.01	−0.84/−0.04/0.20	1/1/1	8/9/9
EMAQ11	5.42/5.41/5.23	5.00/5.00/5.00	1.24/1.37/1.34	0.47/−0.13/−0.39	2.70/2.04/2.51	1/1/1	9/9/9
EMAQ12	5.11/5.29/5.22	5.00/5.00/5.00	1.07/1.26/1.24	0.14/−0.01/−0.12	6.06/3.42/3.93	1/1/1	9/9/9
EMAQ13	5.93/5.24/5.19	6.00/5.00/5.00	2.01/1.96/1.85	−0.33/−0.10/0.00	−0.22/−0.08/0.15	1/1/1	9/9/9
EMAQ14	5.40/5.23/5.23	5.00/5.00/5.00	1.27/1.26/1.28	0.14/−0.18/−0.52	2.79/3.00/2.97	1/1/1	9/9/9
EMAQ15	4.81/4.52/4.83	5.00/4.00/5.00	2.52/2.38/2.39	0.05/0.25/0.08	−1.07/−0.87/−0.91	1/1/1	9/9/9
EMAQ16	4.06/3.85/4.09	4.00/4.00/4.00	2.14/2.18/2.21	0.29/0.50/0.37	−0.49/−0.35/−0.47	1/1/1	9/9/9
EMAQ17	3.18/3.35/3.38	3.00/3.00/3.00	2.05/2.15/2.16	0.86/0.73/0.74	0.19/−0.07/−0.02	1/1/1	9/9/9
EMAQ18	5.07/5.04/4.84	5.00/5.00/5.00	1.55/1.64/1.66	−0.25/−0.26/−0.31	1.73/1.10/1.18	1/1/1	9/9/9
EMAQ19	4.50/4.27/4.30	5.00/5.00/5.00	2.28/2.29/2.37	0.14/0.18/0.25	−0.59/−0.62/−0.71	1/1/1	9/9/9
EMAQ20	5.14/4.96/4.97	5.00/5.00/5.00	1.42/1.58/1.53	−0.16/−0.34/−0.34	1.63/0.99/1.18	1/1/1	9/9/9
EMAQ21	4.45/4.55/4.81	5.00/5.00/5.00	1.79/1.82/2.02	0.00/0.14/0.07	0.35/0.51/−0.12	1/1/1	9/9/9
EMAQ22	5.65/5.56/5.62	5.00/5.00/5.00	1.19/1.23/1.27	1.28/0.13/0.37	1.31/2.83/2.28	3/1/1	9/9/9

**Note:**

# Sample 1: *n* = 323, 18–24 years old, before the pandemic; Sample 2: *n* = 1,011, 18–24 years old, after the start of the pandemic; Sample 3: *n* = 909, 25 years or older, after the start of the pandemic.

Exploratory factor analysis indicated, for the three samples, that the retention of two factors would be the most appropriate (see Table S1–S3 for complete results of the factorial extractions of two and four factors for each sample). Information on the EMAQ initial and refined two-factor models for each sample is presented in Table 4.

**Table 4:** Factorial models for the Emotional Appetite Questionnaire (EMAQ) estimated by exploratory factor analysis for samples before (Sample 1) and during the pandemic (Samples 2 and 3).

Exploratory Factor Analysis (EFA)	Sample 1 (*n* = 323) 18–24 years old				Sample 2 (*n* = 1,011) 18–24 years old				Sample 3 (*n* = 909) 25 years old or older			
**Model factorability**	**initial** ^#^		**refined** ^##^		**initial** ^#^		**refined** ^##^		**initial**		**refined** ^##^	
KMO	0.830 [0.832–0.842]		0.845 [0.846–0.863]		0.883 [0.881–0.896]		0.882 [0.877–0.893]		0.872 [0.867–0.884]		0.875 [0.868–0.893]	
**Model fit**												
CFI	0.982 [0.984–0.984]		0.981 [0.981–0.988]		0.953 [0.943–0.969]		0.953 [0.940–0.968]		0.959 [0.952–0.974]		0.955 [0.943–0.968]	
TLI	0.978 [0.979–0.980]		0.975 [0.974–0.984]		0.940 [0.928–0.961]		0.938 [0.921–0.958]		0.949 [0.939–0.968]		0.941 [0.925–0.958]	
RMSEA	0.052 [0.043–0.061]		0.027 [0.006–0.041]		0.068 [0.060–0.072]		0.078 [0.066–0.085]		0.067 [0.058-–0.070]		0.079 [0.069–0.086]	
**Unidimensionality assessment**												
UniCo	0.701 [0.650–0.762]		0.752 [0.673–0.844]		0.830 [0.778–0.884]		0.866 [0.820–0.915]		0.789 [0.735–0.836]		0.828 [0.764–0.874]	
ECV	0.644 [0.597–0.710]		0.662 [0.600–0.727]		0.725 [0.693–0.755]		0.731 [0.704–0.765]		0.693 [0.660–0.728]		0.696 [0.669–0.731]	
MiReal	0.295 [0.243–0.326]		0.319 [0.280–0.362]		0.290 [0.264–0.307]		0.299 [0.275–0.320]		0.307 [0.288–0.328]		0.328 [0.304–0.344]	
**EFA—2-factor**												
***Emotions***	F1	F2	F1	F2	F1	F2	F1	F2	F1	F2	F1	F2
1. Sad	0.656	–	0.656	–	0.674	–	0.662	–	0.713	–	0.720	–
2. Bored	0.642	–	0.643	–	0.618	–	0.616	–	0.740	–	0.747	–
3. Confident	–	0.614	–	0.627	–	0.633	–	0.639	–	0.729	–	0.732
4. Angry	0.440	–	0.468	–	0.563	–	0.558	–	0.601	–	0.606	–
5. Anxious	0.553	–	0.450	–	0.595	–	0.576	–	0.664	–	0.661	–
6. Happy	–	0.699	–	0.686	–	0.758	–	0.755	–	0.789	–	0.785
7. Frustrated	0.581	–	0.563	–	0.608	–	0.586	–	0.635	–	0.636	–
8. Tired	0.319	–	–	–	0.463	–	–	–	0.449	–	–	–
9. Depressed	0.666	–	0.649	–	0.709	–	0.676	–	0.656	–	0.640	–
10. Frightened	–	–	–	–	–	–	–	–	–	–	–	–
11. Relaxed	–	0.571	–	0.580	–	0.549	–	0.551	–	0.554	–	0.545
12. Playful	–	0.698	–	0.696	–	0.657	–	0.663	–	0.682	–	0.677
13. Lonely	0.367	–	–	–	0.426	–	–	–	0.427	–	–	–
14. Enthusiastic	–	0.732	–	0.740	–	0.731	–	0.729	–	0.809	–	0.823
***Situations*** ^£^												
15. Pressure	0.686	–	0.689	–	0.640	–	0.639	–	0.680	–	0.678	–
16. Discussion	0.765	–	0.784	–	0.757	–	0.781	–	0.797	–	0.805	–
17. Tragedy	0.693	–	0.719	–	0.730	–	0.757	–	0.672	–	0.670	–
18. In love	0.324	0.359	–	–	–	0.387	–	–	–	0.313	–	–
19. End of relationship	0.582	–	0.579	–	0.547	–	0.540	–	0.556	–	0.553	–
20. Hobby	–	0.494	–	0.491	–	0.444	–	0.401	–	0.389	–	0.365
21. Lost property/money	0.461	–	0.459	–	0.606	–	0.619	–	0.583	–	0.582	–
22. Good news	–	–	–	–	–	–	–	–	–	–	–	–
**Average Variance Extracted (AVE)**	0.306	0.331	0.345	0.382	0.339	0.369	0.369	0.409	0.358	0.378	0.392	0.427
**Correlation factors (F1 × F2)**	−0.174		−0.206		−0.345		−0.337		−0.290		−0.288	
**H index (factor stability)**	0.891	0.834	0.888	0.829	0.903	0.842	0.899	0.839	0.912	0.870	0.909	0.869

**Notes:**

# Initial model: removal of items 10 and 22.

## Refined model: deleted items 8, 10, 13, 18 and 22; KMO, Kaiser-Meyer- Olkin; CFI, Comparative Fit Index; TLI, *T*ucker-Lewis Index; RMSEA, Root Mean Square Error of Approximation; UniCo, unidimensional Congruence; ECV, Explained Common Variance; MiReal = Mean of Absolute Residual Item loading.

£ Expressions related to situations have been abbreviated. For the original, see Table 1. Values in square brackets represent the 95% confidence interval for *bootstrap* resamplings.

It was observed that the factorial solution of the three samples was practically identical, both in the number of factors to be retained and in the allocation of items within these factors. Thus, factor 1 (F1) grouped items that represented emotions and situations with negative valence, while factor 2 (F2) concentrated items with positive valence. There was a need to refine the models by the exclusion of items 8 (Tired), 13 (Lonely), and 18 (In love), which had low factor loadings (fitted model).

According to UniCo, ECV, and MiReal, there was no evidence that a one-dimensional matrix supports the EMAQ data for the samples. The stability of the factors and the fit of the models were considered adequate. Thus, it is concluded that the data obtained with the EMAQ for the three samples were fitted to the two-factor model proposal after refinement and validity is demonstrated with respect to the internal structure of the instrument.

The validity of the convergent construct was limited in all samples (VEM = 0.345–0.427). The discriminant validity of the factors, in turn, was adequate (VEM = 0.345–0.427; r² = 0.042–0.114), as was the reliability of the data obtained (Sample 1: CC = 0.79–0.85 and α_ord_ = 0.78–0.85; Sample 2: CC = 0.82–0.87 and α_ord_ = 0.80–0.86; Sample 3: CC = 0.80–0.88 and α_ord_ = 0.80–0.87).

Strict invariance of the refined EMAQ models was shown between the samples (ΔCFI_M1–M0_ = −0.004; ΔCFI_M2–M1_ = 0.001; ΔCFI_M3–M2_ < 0.001), allowing comparisons between them.

Regarding the validity of the EMAQ against external measures, the correlations between the Negative Emotions and Situations factor of the EMAQ and the Emotional Eating factor of the TFEQ-18 were strong and highly significant (r = 0.84 to 0.92; *p* < 0.001) which indicates close proximity between the constructs. For the Positive Emotions and Situations factor, the correlation was weak and negative, as expected (r = −0.13 to −0.30; *p* = 0.001–0.085).

There was a higher prevalence of people who reported increasing their appetite in the face of positive emotions/situations (Sample 1 = 52.0% [CI_95%_: 46.5–57.5]; Sample 2 = 57.5 [CI_95%_: 54.5–60.5]; Sample 3 = 52.3% [CI_95%_: 49.1–55.5]). Regarding negative emotions/situations, a significant prevalence of both an increase (Sample 1 = 54.8% [CI_95%_: 49.4–60.2]; Sample 2 = 50.0% [CI_95%_: 46. 9–53.1]; Sample 3 = 56.2% [CI_95%_: 53.0–59.4]) and a decrease (Sample 1 = 38.4% [CI_95%_: 33.1–43, 7]; Sample 2 = 44.5% [CI_95%_: 41.4–47.6]; Sample 3 = 35.4% [CI_95%_: 32.3–38.5]) of appetite was found.

The prevalence of reduced, unchanged, and increased appetite considering each item of the EMAQ refined model and the comparison of emotional appetite between the samples are shown in Table 5.

**Table 5:** Prevalence (P) and 95% confidence interval (95% CI) of decreased, unchanged, and increased appetite for each item of the refined EMAQ model in the different samples.

Item/Category^§^	^§§^Sample 1		Sample 2		Sample 3		z test		
	***p***	**CI**_**95%**_	***p***	**CI**_**95%**_	***p***	**CI**_**95%**_	***p***_1×2_	***p***_2×3_	***p***_1×3_
**Emotions**									
**1. Sad**									
Decreases	38.7	[33.4–44.0]	42.9	[39.8–46.0]	37.7	[34.5–40.9]	0.183	**0.020**	0.750
Does not change	13.9	[10.1–17.7]	15.1	[12.9–17.3]	18.9	[16.4–21.4]	0.627	**0.023**	**0.043**
Increases	47.4	[41.9–52.9]	42.0	[39.0–45.0]	43.4	[40.2–46.6]	0.088	0.565	0.203
**2. Bored**									
Decreases	38.4	[33.1–43.7]	35.8	[32.8–38.8]	28.4	[25.5–31.3]	0.398	**0.001**	**0.001**
Does not change	25.1	[20.4–29.8]	29.0	[26.2–31.8]	30.0	[27.0–33.0]	0.174	0.631	0.095
Increases	36.5	[31.2–41.8]	35.2	[32.3–38.1]	41.6	[38.4–44.8]	0.671	**0.004**	0.108
**3. Confident**									
Decreases	12.1	[8.5–15.7]	15.3	[13.1–17.5]	14.5	[12.2–16.8]	0.155	0.623	0.284
Does not change	71.8	[66.9–76.7]	66.0	[63.1–68.9]	65.7	[62.6–68.8]	0.053	0.890	**0.045**
Increases	16.1	[12.1–20.1]	18.7	[16.3–21.1]	19.8	[17.2–22.4]	0.290	0.541	0.144
**4. Angry**									
Decreases	35.3	[30.1–40.5]	35.1	[32.2–38.0]	28.4	[25.5–31.3]	0.948	**0.002**	**0.020**
Does not change	36.2	[31.0–41.4]	37.1	[34.1–40.1]	43.6	[40.4–46.8]	0.770	**0.004**	**0.020**
Increase	28.5	[23.6–33.4]	27.8	[25.0–30.6]	28.0	[25.1–30.9]	0.807	0.884	0.891
**5. Anxious**									
Decreases	19.5	[15.2–23.8]	25.1	[22.4–27.8]	18.7	[16.2–21.2]	**0.043**	**0.001**	0.752
Does not change	9.6	[6.4–12.8]	6.7	[5.2–8.2]	8.4	[6.6–10.2]	0.083	0.158	0.511
Increases	70.9	[65.9–75.9]	68.2	[65.3–71.1]	72.9	[70.0–75.8]	0.362	**0.024**	0.490
**6. Happy**									
Decreases	5.3	[2.9–7.7]	8.1	[6.4–9.8]	8.1	[6.3–9.9]	0.095	1,000	0.098
Does not change	55.7	[50.3–61.1]	49.1	[46.0–52.2]	51.7	[48.4–55.0]	**0.039**	0.255	0.216
Increases	39.0	[33.7–44.3]	42.8	[39.7–45.9]	40.2	[37.0–43.4]	0.228	0.248	0.705
**7. Frustrated**									
Decreases	32.5	[27.4–37.6]	30.9	[28.1–33.7]	24.0	[21.2–26.8]	0.589	**0.001**	**0.003**
Does not change	26.0	[21.2–30.8]	24.1	[21.5–26.7]	27.7	[24.8–30.6]	0.490	0.072	0.556
Increases	41.5	[36.1–46.9]	45.0	[41.9–48.1]	48.3	[45.0–51.6]	0.270	0.148	**0.035**
**9. Depressed**									
Decreases	45.5	[40.1–50.9]	52.6	[49.5–55.7]	44.1	[40.9–47.3]	**0.026**	**<0.001**	0.664
Does not change	16.7	[12.6–20.8]	13.9	[11.8–16.0]	19.3	[16.7–21.9]	0.198	**0.001**	0.302
Increases	37.8	[32.5–43.1]	33.5	[30.6–36.4]	36.6	[33.5–39.7]	0.157	0.155	0.701
**11. Relaxed**									
Decreases	5.6	[3.1–8.1]	10.4	[8.5–12.3]	12.3	[10.2–14.4]	**0.009**	0.189	**0.001**
Does not change	67.5	[62.4–72.6]	54.8	[51.7–57.9]	57.4	[54.2–60.6]	**<0.001**	0.252	**0.001**
Increases	26.9	[22.1–31.7]	34.8	[31.9–37.7]	30.3	[27.3–33.3]	**0.009**	**0.036**	0.249
**12. Playful**									
Decreases	8.0	[5.0–11.0]	8.9	[7.1–10.7]	8.7	[6.9–10.5]	0.617	0.877	0.699
Does not change	77.1	[72.5–81.7]	64.2	[61.2–67.2]	67.9	[64.9–70.9]	**<0.001**	0.088	**0.002**
Increases	14.9	[11.0–18.8]	26.9	[24.2–29.6]	23.4	[20.6–26.2]	**<0.001**	0.078	**0.001**
**14. Enthusiastic**									
Decreases	6.5	[3.8–9.2]	11.2	[9.3–13.1]	11.1	[9.1–13.1]	**0.015**	0.945	**0.017**
Does not change	64.1	[58.9–69.3]	60.8	[57.8–63.8]	59.4	[56.2–62.6]	0.288	0.532	0.138
Increases	29.4	[24.4–34.4]	28.0	[25.2–30.8]	29.5	[26.5–32.5]	0.627	0.468	0.973
**Situations**									
**15. When under pressure**									
Decreases	45.2	[39.8–50.6]	51.4	[48.3–54.5]	42.8	[39.6–46.0]	0.056	**<0.001**	0.455
does not change	17.0	[12.9–21.1]	15.3	[13.1–17.5]	19.5	[16.9–22.1]	0.465	**0.015**	0.324
Increases	37.8	[32.5–43.1]	33.3	[30.4–36.2]	37.7	[34.5–40.9]	0.138	**0.044**	0.975
**16. After a heated argument**									
Decreases	54.5	[49.1–59.9]	57.4	[54.4–60.4]	52.7	[49.5–55.9]	0.360	**0.039**	0.578
Does not change	26.0	[21.2–30.8]	25.5	[22.8–28.2]	27.2	[24.3–30.1]	0.858	0.398	0.676
Increases	19.5	[15.2–23.8]	17.1	[14.8–19.4]	20.1	[17.5–22.7]	0.325	0.091	0.817
**17. After a tragedy with someone close**									
Decreases	71.5	[66.6–76.4]	66.7	[63.8–69.6]	65.5	[62.4–68.6]	0.108	0.611	0.052
Does not change	18.3	[14.1–22.5]	22.0	[19.4–24.6]	22.7	[20.0–25.4]	0.146	0.753	0.099
Increases	10.2	[6.9–13.5]	11.3	[9.3–13.3]	11.8	[9.7–13.9]	0.583	0.732	0.437
**19. After ending a relationship**									
Decreases	41.5	[36.1–46.9]	43.7	[40.6–46.8]	46.9	[43.7–50.1]	0.487	0.160	0.094
Does not change	33.7	[28.5–38.9]	34.8	[31.9–37.7]	29.2	[26.2–32.2]	0.717	**0.010**	0.140
Increases	24.8	[20.1–29.5]	21.5	[19.0–24.0]	23.9	[21.1–26.7]	0.215	0.210	0.745
**20. When engaged in an enjoyable hobby**									
Decreases	17.6	[13.4–21.8]	24.0	[21.4–26.6]	23.2	[20.5–25.9]	**0.016**	0.680	**0.036**
Does not change	54.2	[48.8–59.6]	48.0	[44.9–51.1]	49.7	[46.4–53.0]	0.052	0.457	0.165
Increases	28.2	[23.3–33.1]	28.0	[25.2–30.8]	27.1	[24.2–30.0]	0.944	0.659	0.703
**21. After losing money or property**									
Decreases	43.0	[37.6–48.4]	39.2	[36.2–42.2]	34.1	[31.0–37.2]	0.225	**0.021**	**0.004**
Does not change	40.6	[35.2–46.0]	43.2	[40.1–46.3]	39.6	[36.4–42.8]	0.411	0.110	0.753
Increases	16.4	[12.4–20.4]	17.6	[15.3–19.9]	26.3	[23.4–29.2]	0.620	**<0.001**	**<0.001**

**Notes:**

§ excluded items: 8, 10, 13, 18, and 22 (refined model).

§§ Sample 1: *n* = 323, 18–24 years old, before the pandemic; Sample 2: *n* = 1, 011, 18–24 years old, after the beginning of the pandemic; Sample 3: *n* = 909, ≥25 years old, after the beginning of the pandemic. Values in bold indicate statistical significance (*p* < 0.05).

In the three samples, there was a significant increase in appetite in the face of anxiety and a decrease in appetite in the face of tragedy.

When comparing the samples, it was generally noted that when negative emotions and situations (bored, angry, anxious, frustrated, depressed, and under pressure) were present, the proportion of individuals with decreased appetite was lower in Sample 3 (older and during the pandemic) compared to the others. The positive emotions Relaxed and Enthusiastic were found to decrease the appetite more often in samples 2 and 3 (during the pandemic) than in Sample 1 (before the pandemic).

For the negative emotions and situations Bored, Anxious, and Losing money/property, there was a higher prevalence of increase in appetite among individuals in Sample 3 in relation to the other samples. For the emotions Relaxed and Playful, the higher proportion of increased appetite in Samples 2 and 3 stands out.

**Figure 1:**
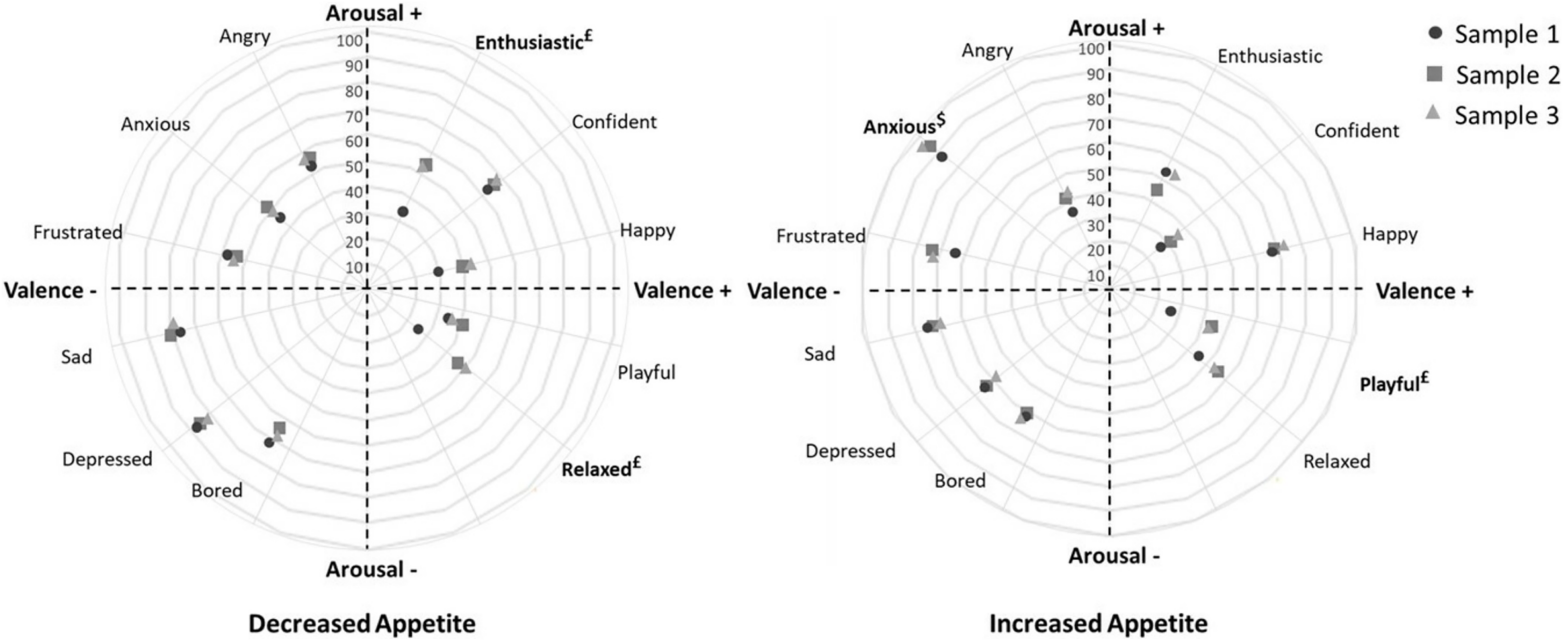
Circumplex model showing the prevalence of appetite changes in participants from the three samples in relation to different emotions. Left panel: individuals who decreased food consumption in the face of negative emotions (*n*_sample1_ = 124; *n*_sample2_ = 450; *n*_sample3_ = 322) and positive emotions (*n*_sample1_ = 50; *n*_sample2_ = 176; *n*_sample3_ = 166); Right panel: individuals who increased their food consumption in the face of negative emotions (*n*_sample1_ = 177; *n*_sample2_ = 505; *n*_sample3_ = 511) and positive emotions (*n*_sample1_ = 168; *n*_sample2_ = 581; *n*_sample3_ = 475). ^£^Significant difference between samples 1 and 2, and 1 and 3 (z test; *p* < 0.05); ^$^Significant difference between samples 1 and 3 (z test; *p* < 0.05).

Figure 1 shows the circumplex model designed to represent the prevalence of appetite changes in participants from the three samples when faced with different emotions.

Emotions with negative valence and activation (sad, bored, and depressed) contributed more to appetite reduction. Emotions with negative valence (except “angry”) were most important in increasing appetite, regardless of the level of activation. The important role of anxiety in increasing appetite is highlighted. Among the positive emotions associated with increased appetite, feelings of happiness stand out, and decreased appetite when individuals feeling confident.

In the context of appetite reduction, Sample 1 (pre-pandemic) differed significantly from the others in terms of enthusiastic (*p* = 0.013–0.018) and relaxed (*p* = 0.002–0.009) feelings. Regarding increased appetite, there was a difference between samples in relation to anxiety. In Sample 3 (during the pandemic and in older participants), the prevalence of increased appetite was higher than in Sample 1 (*p* = 0.001). Sample 1 (pre-pandemic) also had a lower prevalence of people reporting increased food consumption than the other samples (*p* < 0.001) when feeling “playful”.

## Discussion

The present study adapted the EMAQ into Portuguese considering different Lusophone contexts. The data obtained with this version of the EMAQ were valid and reliable when applied to three samples of Brazilian adults in the context before and after the COVID-19 pandemic onset. The two-factor model (emotions and situations: 1. Negative; 2. Positive) was the most appropriate for the samples (model that presented the best psychometric indicators). Similarly, both Bourdier et al. (2017) in a French sample (2017) and Sabry et al. (2020) in a Brazilian sample showed that the two-factor model of the EMAQ, which primarily considers the valence of emotions/situations, had a better fit.

The low factor loading of some items (*e.g*., tired, lonely, and when falling in love) found in our study suggests that some emotions/situations may not be interpreted by young adults as interfering with their eating habits. It may be associated with sample characteristics and/or the context of data collection. Interestingly, another study (Bourdier et al., 2017) with young adults showed a similar profile of lower factor loadings for the same items. These findings reinforce the importance of testing the psychometric properties of the data collected with a given instrument when there are changes in the sample or context, to obtain more accurate results.

The correlations between the EMAQ factors and the Emotional Eating factor of the TFEQ (external measure) support the validity of the data obtained with this instrument in the study samples. The high correlation between F1 (negative emotions/situations) and emotional eating is justified by the convergence between the concepts assessed (Geliebter & Aversa, 2003; Karlsson et al., 2000), while the weak relationship with the positive factor (F2) is related to the fact that the TFEQ only considers negative emotional aspects. Barnhart, Braden & Price (2021) found that difficulties in emotion control were related to eating processes with negative valence (appetite and eating) but not to processes with positive valence, which could explain our results.

The second hypothesis tested in our work deals with the influence of positive and negative emotions/situations on food appetite. Psychobiology has studied the distinction between the brain mechanisms responsible for physiological hunger and hedonic hunger or appetite (Berthoud, Münzberg & Morrison, 2017). The former reflects a physical need for nutrients and the latter takes into account other stimuli (sensory, emotional, cost, and availability) that may trigger the desire to consume a particular food. These stimuli include the experience of emotions/situations that can promote or suppress appetite (Macht, 2008; Sabry et al., 2020). Thus, when confronted with different emotions/situations, the person may experience a greater or lesser desire for food, which may effectively increase or decrease their food consumption. This is due to the integration of different brain pathways that allow the connection between the biological and psychological aspects of eating (Berthoud, Münzberg & Morrison, 2017). This idea reinforces the theory that eating isn’t limited to biological nutrition and emphasizes the importance of considering individual perceptions of one’s behavior in order to promote change and avoid imbalances.

In the present study, it was found that the increase in food consumption was more meaningful for positive emotions, while for negative emotions, both the increase and decrease in consumption were relevant. The circumplex model was used to illustrate the contribution of each emotion to the decrease or increase in appetite. Negative high-activation emotions contributed to increase emotional appetite and negative and low-activation emotions to reduce appetite. In the context of the COVID-19 pandemic, it was found that participants’ emotional appetite decreased significantly when they felt relaxed, with no significant changes in the face of other emotions and situations.

Changes in food consumption when facing negative emotions and situations indicate two profiles of individuals according to Macht’s theory (Macht, 2008). The first refers to individuals who suppress their appetite. The decrease in appetite in these cases can be explained by the high intensity of negative emotions, which can limit routine activities in a process of behavioral deactivation (Macht, 2008). The second profile includes people who have an increased appetite, which may be related to the high activation that leads to food cravings (usually hyper-palatable food) to relieve negative emotions (Canetti, Bachar & Berry, 2002; Macht, 2008). In the present study, a significant prevalence of the two aforementioned profiles was found, a result that differs from that of Sabry et al. (2020), who found a higher prevalence of appetite reduction in negative contexts. This difference could be related to the characteristics of the samples studied, because the authors (Sabry et al., 2020) studied a population with a higher mean age (women in the Unified Health System, age: 33.6 ± 8.9 years) than the samples in this study (age: 19.7 ± 1.5 to 28.9 ± 3.1 years). These differences between results highlight that emotional appetite have specific characteristics for different groups. Therefore, applying the EMAQ to samples with different characteristics may help to understand emotional appetite in a way that focuses more on the vulnerabilities of each group.

The inclusion of the emotion activation component (Russell, 1980) in the assessment of emotional appetite based on the circumplex model brought to light some peculiarities that could not be determined with valence alone. The results obtained are consistent with Macht’s theory (Macht, 2008), which assumes that intensity, preexisting eating habits, and specificity of emotions can determine consumption. Thus, the fact that emotions with negative valence and activation (sad, bored, and depressed) contribute most to appetite reduction can be explained by a specific modulation effect, in which these emotions elicit a physiological response consistent with their lower activation, thus reducing appetite (Macht, 2008). On the other hand, discomfort in emotionally stressful situations (*e.g*., high activation of anxiety) may promote cognitive deconstruction, in which the person focus attention on simpler rational processes and avoid contact with his or her more complex emotions and feelings in order to reduce discomfort (Canetti, Bachar & Berry, 2002; Heatherton & Baumeister, 1991). In this process, the constraints imposed on eating may be temporarily inhibited, which promotes an increase in appetite and eating, providing short-term relief from the aversive state (Heatherton & Baumeister, 1991). In addition, Kaplan & Kaplan (1957) mention that some people have difficulty distinguishing between internal signals (*e.g*., hunger and satiety) and emotions, so affective states can also cause an increase in appetite.

The fact that the reduction in appetite was higher in subjects in samples 2 and 3 (during the pandemic) when experiencing positive emotions opens space for reflection on the pandemic scenario and the eating process. The restriction of personal contacts during the pandemic could be related to this change in appetite, as eating due to positive emotions has been associated with socialization processes, such as celebrations or meetings, and not only with positive emotions *per se* (Alvarenga, 2015; Frayn & Knäuper, 2018; Macht, 1999). Regarding the effects of negative emotions on eating behavior during the pandemic, studies conducted before (Bourdier et al., 2017; Evers et al., 2013; Lindeman & Stark, 2001; Sabry et al., 2020; Van Strien et al., 2013) and during this period (Barnhart, Braden & Price, 2021; Mason, Barrington-Trimis & Leventhal, 2021; Poelman et al., 2021) have pointed to important changes in food consumption, such as increases in the consumption of hyperpalatable foods and episodes of binge eating. However, in the present study, no significant changes in emotional appetite were found in the period before and during the pandemic. It should be noted, however, that there was no direct assessment of food consumption but an examination of individual perceptions of behavior. Thus, it is possible that food consumption itself changed during the pandemic, although emotional appetite (the volitional aspect of attitudes (Alvarenga & Koritar, 2019)) remained stable.

It is also worth noting that despite the subtle differences in emotional appetite observed between samples, there was generally some stability in the evaluation of this concept between samples. This may be because the samples were recruited from the general population (*i.e*., without eating disorders), so the EMAQ responses tended to show unchanged appetite, with slight fluctuations toward decreases or increases. Thus, further studies using the EMAQ in people with eating disorders may be relevant to expand the evidence and improve the toolkit of nutrition and mental health professionals to understand emotional appetite as a possible predictor of dysfunctional eating behaviors.

The present study has some limitations, such as the cross-sectional design, which does not allow the determination of cause-effect relationships, and the use of a convenience sample. This sampling design limits the generalization of the results, since students with specific characteristics (based on the convenience and context of the researchers) were included in Study 1, while in Study 2, online data collection favored the participation of people with internet access and digital proficiency. It compromises the representativeness of the sample, since Brazil is a country marked by large socioeconomic inequalities and inequalities in access to technology, which can limit participation to the population with higher education and income. Nevertheless, we believe that this study adds evidence to the topic of emotional appetite which may be relevant for health professionals (mainly nutritionists and psychologists). It also starts and encourages a discussion about the influence of emotions on the eating behavior of a group of Brazilians. The use of two methods of data collection (paper and online) wasn’t a limitation because the EMAQ performed the same in both application formats, which was confirmed by the between-sample analysis of invariance. It should also be mentioned that it is not possible to state with certainty that the COVID-19 pandemic was associated with the increase/decrease in food consumption. The EMAQ is not specific for to the pandemic and the consumption assessed may not only be associate with negative emotions caused by the pandemic, but also with changes in the access to ready-to-eat foods or increased time to cook food due to having more time due to remote work, *etc*. Thus, we understand that this is a limitation of this study and that conclusions should be made with caution.

With this study, we aimed to expand the understanding of emotional appetite by using the circumplex model to study emotions, which captures not only their valence but also their activation. In addition, the validity and reliability of the data collected using the EMAQ was examined to ensure the quality of the data presented. It was also found that young adults’ emotional appetite differs for certain emotions (*e.g*., anxiety, playful, excited, relaxed) but generally remains stable across different age groups and contexts.

## Conclusion

The adaptation of the Portuguese version of the Emotional Appetite Questionnaire (EMAQ) worked similarly before and after the COVID-19 pandemic when applied to Brazilian adults. The data obtained were valid and reliable and the two-factor model was the most appropriate. Overall, there were no major changes in emotional appetite after the pandemic outbreak. Positive emotions/situations were more likely to increase appetite, while negative emotions/situations were more likely to both decrease and increase appetite. Negative emotions and low activation were relevant to appetite reduction, while anxiety (high activation) contributed significantly to appetite increase. Therefore, the assessment of emotional appetite considering not only the valence of emotions, but also their activation (circumplex model) becomes relevant in the management of eating behavior.

## Supplemental Information

10.7717/peerj.14597/supp-1 Supplemental Information 1 Supplementary tables with complete evaluation of four- and two-factor models of EMAQ for each sample.

10.7717/peerj.14597/supp-2 Supplemental Information 2 Dataset. Demographic characteristics, the answers given to the EMAQ (in the general database tab), and the raw EMAQ and TFEQ data used to estimate the polychoric correlation between the scales.

10.7717/peerj.14597/supp-3 Supplemental Information 3 R Codes for EMAQ data.

10.7717/peerj.14597/supp-4 Supplemental Information 4 Emotional Appetite Questionnaire.

## Additional Information and Declarations

### Data Availability

The following information was supplied regarding data availability: The raw data is available in the Supplemental File.
