# Supplementary material for: Emotional Appetite Questionnaire: psychometric properties in Brazilian adult samples before and after the COVID-19 pandemic onset
Source: PeerJ. 2023 Jan 26;11:e14597. doi: 10.7717/peerj.14597 (PMC9884477; doi:10.7717/peerj.14597)
Supplement: Supplemental Information 1 [file peerj-11-14597-s001.docx]

# Supplementary Material

**Supplemental Table S1.** Four- and two-factor models of the Emotional Appetite Questionnaire (EMAQ) analyzed by exploratory factor analysis for sample of adults before the pandemic (n=323).

| **Exploratory Factor Analysis (EFA)** | **Sample 1 (n=323)**  **18-24 years old** | | | | **Sample 1 (n=323)**  **18-24 years old** | |
| --- | --- | --- | --- | --- | --- | --- |
| **Model factorability** |  | | | |  | |
| KMO | 0.830 ]0.835-0.850[ | | | | 0.830 ]0.832-0.842[ | |
| **Model fit** |  | | | |  | |
| CFI | 0.998 ]0.997-0.997[ | | | | 0.982 ]0.984-0.984[ | |
| TLI | 0.997 ]0.995-0.995[ | | | | 0.978 ]0.979-0.980[ | |
| RMSEA | 0.012 ]0.003-0.021[ | | | | 0.052 ]0.043-0.061[ | |
| **Unidimensionality Assessment** |  | | | |  | |
| UniCo | 0.701 ]0.653-0.769[ | | | | 0.701 ]0.650-0.762[ | |
| ECV | 0.644 ]0.591-0.708[ | | | | 0.644 ]0.597-0.710[ | |
| MiReal | 0.295 ]0.251-0.326[ | | | | 0.295 ]0.243-0.326[ | |
|  |  | | | |  | |
| **EFA** | **Four-factor model^#^** | | | | **Two-factor model^#^** | |
|  | **F1** | **F2** | **F3** | **F4** | **F1** | **F2** |
| ***Emotions*** |  |  |  |  |  |  |
| 1. Sad | 0.835 | - | - | - | 0.656 | - |
| 2. Bored | 0.581 | - | - | - | 0.642 | - |
| 3. Confident | - | 0.631 | - | - | - | 0.614 |
| 4. Angry | - | - | - | 0.543 | 0.440 | - |
| 5. Anxious | - | - | 0.776 | - | 0.553 | - |
| 6. Happy | - | 0.719 | - | - | - | 0.699 |
| 7. Frustrated | - | - | - | 0.313 | 0.581 | - |
| 8. Tired | - | - | - | 0.330 | 0.319 | - |
| 9. Depressed | 0.614 | - | - | - | 0.666 | - |
| 10. Frightened | - | - | - | - | - | - |
| 11. Relaxed | - | 0.572 | - | - | - | 0.571 |
| 12. Playful | - | 0.672 | - | - | - | 0.698 |
| 13. Lonely | - | - | - | - | 0.367 | - |
| 14. Enthusiastic | - | 0.717 | - | - | - | 0.732 |
| ***Situations ^£^*** |  |  |  |  |  |  |
| 15. Pressure | - | - | 0.332 | 0.522 | 0.686 | - |
| 16. Discussion | - | - | - | 0.788 | 0.765 | - |
| 17. Tragedy | - | - | - | 0.572 | 0.693 | - |
| 18. In love | - | 0.381 | - | - | 0.324 | 0.359 |
| 19. End of relationship | 0.369 | - | - | 0.306 | 0.582 | - |
| 20. Hobby | - | 0.495 | - | - | - | 0.494 |
| 21. Lost property/money | - | - | - | 0.457 | 0.461 | - |
| 22. Good news | - | - | - | - | - | - |
| **H index (factor stability)** | 0.834 | 0.836 | 0.740 | 0.834 | 0.891 | 0.834 |

^#^Initial model: removal of items 10 and 22; KMO = *Kaiser-Meyer- Olkin*; CFI = *Comparative Fit Index*; TLI = *Tucker-Lewis Index*; RMSEA = *Root Mean Square Error of Approximation*; UniCo = *unidimensional Congruence*; ECV = *Explained Common Variance*; MiReal = *Mean of Absolute Residual Item loading*; **^£^**Expressions related to situations have been abbreviated. For the original, see Table 1 in article. Values in square brackets represent the 95% confidence interval for *bootstrap* resamplings.

**Supplemental Table S2.** Four- and two-factor models of the Emotional Appetite Questionnaire (EMAQ) analyzed by exploratory factor analysis for sample of adults after start of pandemic (n=1.011).

| **Exploratory Factor Analysis (EFA)** | **Sample 2 (n=1,011)**  **18-24 years old** | | | | **Sample 2 (n=1,011)**  **18-24 years old** | |
| --- | --- | --- | --- | --- | --- | --- |
| **Model factorability** |  | | | |  | |
| KMO | 0.883 ]0.880-0.890[ | | | | 0.883 ]0.881-0.896[ | |
| **Model fit** |  | | | |  | |
| CFI | 0.988 ]0.988-0.990[ | | | | 0.953 ]0.943-0.969[ | |
| TLI | 0.980 ]0.980-0.984[ | | | | 0.940 ]0.928-0.961[ | |
| RMSEA | 0.039 ]0.036-0.039[ | | | | 0.068 ]0.060-0.072[ | |
| **Unidimensionality Assessment** |  | | | |  | |
| UniCo | 0.830 ]0.775-0.885[ | | | | 0.830 ]0.778-0.884[ | |
| ECV | 0.725 ]0.691-0.758[ | | | | 0.725 ]0.693-0.755[ | |
| MiReal | 0.290 ]0.263-0.311[ | | | | 0.290 ]0.264-0.307[ | |
|  |  | | | |  | |
| **EFA** | **Four-factor model^#^** | | | | **Two-factor model^#^** | |
|  | **F1** | **F2** | **F3** | **F4** | **F1** | **F2** |
| ***Emotions*** |  |  |  |  |  |  |
| 1. Sad | 0.831 | - | - | - | 0.674 | - |
| 2. Bored | 0.390 | - | 0.401 | - | 0.618 | - |
| 3. Confident | - | 0.611 | - | - | - | 0.633 |
| 4. Angry | - | - | 0.491 | - | 0.563 | - |
| 5. Anxious | 0.331 | - | 0.377 | - | 0.595 | - |
| 6. Happy | - | 0.762 | - | - | - | 0.758 |
| 7. Frustrated | 0.346 | - | 0.454 | - | 0.608 | - |
| 8. Tired | 0.383 | - | - | - | 0.463 | - |
| 9. Depressed | 0.798 | - | - | - | 0.709 | - |
| 10. Frightened | - | - | - | - | - | - |
| 11. Relaxed | - | 0.568 | - | - | - | 0.549 |
| 12. Playful | - | 0.652 | - | - | - | 0.657 |
| 13. Lonely | 0.444 | - | - | - | 0.426 | - |
| 14. Enthusiastic | - | 0.757 | - | - | - | 0.731 |
| ***Situations ^£^*** |  |  |  |  |  |  |
| 15. Pressure | - | - | 0.315 | 0.527 | 0.640 | - |
| 16. Discussion | - | - | - | 0.845 | 0.757 | - |
| 17. Tragedy | - | - | - | 0.804 | 0.730 | - |
| 18. In love | - | 0.405 | - | - | - | 0.387 |
| 19. End of relationship | 0.433 | - | - | 0.337 | 0.547 | - |
| 20. Hobby | - | 0.499 | - | - | - | 0.444 |
| 21. Lost property/money | - | - | - | 0.510 | 0.606 | - |
| 22. Good news | - | - | - | - | - | - |
| **H index (factor stability)** | 0.883 | 0.844 | 0.678 | 0.863 | 0.903 | 0.842 |

^#^Initial model: removal of items 10 and 22; KMO = *Kaiser-Meyer- Olkin*; CFI = *Comparative Fit Index*; TLI = *Tucker-Lewis Index*; RMSEA = *Root Mean Square Error of Approximation*; UniCo = *unidimensional Congruence*; ECV = *Explained Common Variance*; MiReal = *Mean of Absolute Residual Item loading*; **^£^**Expressions related to situations have been abbreviated. For the original, see Table 1 in article. Values in square brackets represent the 95% confidence interval for *bootstrap* resamplings.

**Supplemental Table S3.** Four- and two-factor models of the Emotional Appetite Questionnaire (EMAQ) analyzed by exploratory factor analysis for sample of adults after start of pandemic (n=909).

| **Exploratory Factor Analysis (EFA)** | **Sample 3 (n=909)**  **25 years old or older** | | | | **Sample 3 (n=909)**  **25 years old or older** | |
| --- | --- | --- | --- | --- | --- | --- |
| **Model factorability** |  | | | |  | |
| KMO | 0.872 ]0.868-0.884[ | | | | 0.872 ]0.867-0.884 | |
| **Model fit** |  | | | |  | |
| CFI | 0.987 ]0.985-0.991[ | | | | 0.959 ]0.952-0.974[ | |
| TLI | 0.979 ]0.976-0.985[ | | | | 0.949 ]0.939-0.968[ | |
| RMSEA | 0.043 ]0.038-0.044[ | | | | 0.067 ]0.058-0.070[ | |
| **Unidimensionality Assessment** |  | | | |  | |
| UniCo | 0.789 ]0.736-0.834[ | | | | 0.789 ]0.735-0.836[ | |
| ECV | 0.693 ]0.660-0.721[ | | | | 0.693 ]0.660-0.728[ | |
| MiReal | 0.307 ]0.285-0.327[ | | | | 0.307 ]0.288-0.328[ | |
|  |  | | | |  | |
| **EFA** | **Four-factor model^#^** | | | | **Two-factor model^#^** | |
|  | **F1** | **F2** | **F3** | **F4** | **F1** | **F2** |
| ***Emotions*** |  |  |  |  |  |  |
| 1. Sad | 0.895 | - | - | - | 0.713 | - |
| 2. Bored | 0.839 | - | - | - | 0.740 | - |
| 3. Confident | - | 0.713 | - | - | - | 0.729 |
| 4. Angry | 0.397 | - | - | - | 0.601 | - |
| 5. Anxious | 0.568 | - | - | - | 0.664 | - |
| 6. Happy | - | 0.825 | - | - | - | 0.789 |
| 7. Frustrated | 0.574 | - | - | - | 0.635 | - |
| 8. Tired | - | - | 0.326 | - | 0.449 | - |
| 9. Depressed | 0.668 | - | - | - | 0.656 | - |
| 10. Frightened | - | - | - | - | - | - |
| 11. Relaxed | - | 0.573 | - | - | - | 0.554 |
| 12. Playful | - | 0.678 | - | - | - | 0.682 |
| 13. Lonely | 0.334 | - | - | - | 0.427 | - |
| 14. Enthusiastic | - | 0.813 | - | - | - | 0.809 |
| ***Situations ^£^*** |  |  |  |  |  |  |
| 15. Pressure | - | - | 0.598 | - | 0.680 | - |
| 16. Discussion | - | - | 0.890 | - | 0.797 | - |
| 17. Tragedy | - | - | 0.780 | - | 0.672 | - |
| 18. In love | - | - | - | - | - | 0.313 |
| 19. End of relationship | - | - | 0.458 | - | 0.556 | - |
| 20. Hobby | - | 0.385 | - | - | - | 0.389 |
| 21. Lost property/money | - | - | 0.417 | - | 0.583 | - |
| 22. Good news | - | - | - | - | - | - |
| **H index (factor stability)** | 0.908 | 0.873 | 0.882 | 0.501 | 0.912 | 0.870 |

^#^Initial model: removal of items 10 and 22; KMO = *Kaiser-Meyer- Olkin*; CFI = *Comparative Fit Index*; TLI = *Tucker-Lewis Index*; RMSEA = *Root Mean Square Error of Approximation*; UniCo = *unidimensional Congruence*; ECV = *Explained Common Variance*; MiReal = *Mean of Absolute Residual Item loading*; **^£^**Expressions related to situations have been abbreviated. For the original, see Table 1 in article. Values in square brackets represent the 95% confidence interval for *bootstrap* resamplings.
