# Supplementary material for: Emotional Appetite Questionnaire: psychometric properties in Brazilian adult samples before and after the COVID-19 pandemic onset
Source: PeerJ. 2023 Jan 26;11:e14597. doi: 10.7717/peerj.14597 (PMC9884477; doi:10.7717/peerj.14597)
Supplement: Supplemental Information 4 [file peerj-11-14597-s004.docx]

**Emotional Appetite Questionnaire (EMAQ)**

Instructions: Please tell us first, how your eating behavior is affected by certain emotional states and situations. Mark an X on the scale below. The scale ranges from "1" to "9" where "1" represents a much lower than usual food intake and "9" represents a much higher than usual food intake. The "5" represents the usual food intake.

| **The following refers to EMOTIONS.**  **As compared to usual, do you eat:** | | | | Much Less |  |  |  | Nor less nor more |  |  |  | Much More |
| --- | --- | --- | --- | --- | --- | --- | --- | --- | --- | --- | --- | --- |
|  |  |  |  | 1 | 2 | 3 | 4 | 5 | 6 | 7 | 8 | 9 |
| Quando está: | | | 1. Sad |  |  |  |  |  |  |  |  |  |
|  | |  | 2. Bored |  |  |  |  |  |  |  |  |  |
|  | |  | 3. Confident |  |  |  |  |  |  |  |  |  |
|  | |  | 4. Angry |  |  |  |  |  |  |  |  |  |
|  | |  | 5. Anxious |  |  |  |  |  |  |  |  |  |
|  | |  | 6. Happy |  |  |  |  |  |  |  |  |  |
|  | |  | 7. Frustrated |  |  |  |  |  |  |  |  |  |
|  | |  | 8. Tired |  |  |  |  |  |  |  |  |  |
|  | |  | 9. Depressed |  |  |  |  |  |  |  |  |  |
|  | |  | 10. Frightened |  |  |  |  |  |  |  |  |  |
|  | |  | 11. Relaxed |  |  |  |  |  |  |  |  |  |
|  | |  | 12. Playful |  |  |  |  |  |  |  |  |  |
|  | |  | 13. Lonely |  |  |  |  |  |  |  |  |  |
|  | |  | 14. Enthusiastic |  |  |  |  |  |  |  |  |  |
| **The following refer to SITUATIONS**  As compared to usual, do you eat: | | | | Much Less |  |  |  | Nor less nor more |  |  |  | Much More |
|  |  |  |  | 1 | 2 | 3 | 4 | 5 | 6 | 7 | 8 | 9 |
|  | 15. When under pressure | | |  |  |  |  |  |  |  |  |  |
|  | 16. After a heated argument | | |  |  |  |  |  |  |  |  |  |
|  | 17. After a tragedy of someone close to you | | |  |  |  |  |  |  |  |  |  |
|  | 18. When falling in love | | |  |  |  |  |  |  |  |  |  |
|  | 19. After ending a relationship | | |  |  |  |  |  |  |  |  |  |
|  | 20. When engaged in an enjoyable hobby | | |  |  |  |  |  |  |  |  |  |
|  | 21. After losing money or property | | |  |  |  |  |  |  |  |  |  |
|  | 22. After receiving good news | | |  |  |  |  |  |  |  |  |  |
